# Germline Sequencing of DNA Damage Repair Genes in Two Hereditary Prostate Cancer Cohorts Reveals New Disease Risk-Associated Gene Variants

**DOI:** 10.3390/cancers16132482

**Published:** 2024-07-07

**Authors:** Georgea R. Foley, James R. Marthick, Sionne E. Lucas, Kelsie Raspin, Annette Banks, Janet L. Stanford, Elaine A. Ostrander, Liesel M. FitzGerald, Joanne L. Dickinson

**Affiliations:** 1 Menzies Institute for Medical Research, University of Tasmania, Hobart, TAS 7000, Australia; georgea.foley@utas.edu.au (G.R.F.); james.marthick@utas.edu.au (J.R.M.); sionne.lucas@utas.edu.au (S.E.L.); kelsie.raspin@utas.edu.au (K.R.); annette.banks@utas.edu.au (A.B.); 2 Fred Hutchinson Cancer Center, 1100 Fairview Ave. N., M4-B874, Seattle, WA 98109, USA; jstanfor@fredhutch.org; 3 Cancer Genetics and Comparative Genomics Branch, National Human Genome Research Institute, National Institutes of Health, Bethesda, MD 20892, USA; eostrand@mail.nih.gov

**Keywords:** rare high-risk genes, prostate cancer, DNA repair, genetic susceptibility, germline variants

## Abstract

**Simple Summary:**

An urgent demand exists to identify inherited genetic risk variants for prostate cancer (PrCa), particularly in DNA damage repair genes targetable with precision medicine-based strategies. Though the most heritable common cancer, discovery of rare germline PrCa risk variants is hampered by their low frequency, even in sizeable population datasets. Through utilising two large, independent, familial PrCa resources and their likely enrichment of rare causative variants, we provide robust evidence for several novel risk variants in DNA damage repair genes.

**Abstract:**

Rare, inherited variants in DNA damage repair (DDR) genes have a recognised role in prostate cancer (PrCa) susceptibility. In addition, these genes are therapeutically targetable. While rare variants are informing clinical management in other common cancers, defining the rare disease-associated variants in PrCa has been challenging. Here, whole-genome and -exome sequencing data from two independent, high-risk Australian and North American familial PrCa datasets were interrogated for novel DDR risk variants. Rare DDR gene variants (predicted to be damaging and present in two or more family members) were identified and subsequently genotyped in 1963 individuals (700 familial and 459 sporadic PrCa cases, 482 unaffected relatives, and 322 screened controls), and association analyses accounting for relatedness (M_QLS_) undertaken. In the combined datasets, rare *ERCC3* (rs145201970, *p* = 2.57 × 10^−4^) and *BRIP1* (rs4988345, *p* = 0.025) variants were significantly associated with PrCa risk. A *PARP2* (rs200603922, *p* = 0.028) variant in the Australian dataset and a *MUTYH* (rs36053993, *p* = 0.031) variant in the North American dataset were also associated with risk. Evaluation of clinicopathological characteristics provided no evidence for a younger age or higher-grade disease at diagnosis in variant carriers, which should be taken into consideration when determining genetic screening eligibility criteria for targeted, gene-based treatments in the future. This study adds valuable knowledge to our understanding of PrCa-associated DDR genes, which will underpin effective clinical screening and treatment strategies.

## 1. Introduction

Prostate cancer (PrCa) is responsible for a significant proportion of cancer-related deaths in men worldwide and presents a substantial health burden in terms of morbidity, mental health, and economic costs associated with treatment. Family history is one of the strongest risk factors for PrCa: heritability is estimated at ~57% [1], and both common and rare variants contribute to inherited risk. Notably, a considerable percentage of men with advanced disease harbour clinically actionable germline genetic variants, many of which are aberrations in DNA damage repair (DDR) genes [2,3,4,5,6,7]. Specifically, germline variants in these genes have been observed in 8–16% of metastatic PrCa patients [2,5,8].

Despite recognition of their potential to facilitate early diagnosis and assist in treatment selection, there remains a significant gap in our understanding of the full spectrum of DDR gene variants contributing to PrCa risk [8,9,10,11,12]. Several DDR genes, particularly *BRCA1/2* and *ATM,* have been associated with a substantial increase in disease risk, poorer prognosis, differing responses to treatment, and more aggressive disease [13,14,15,16,17]. Notably, there are many other genes involved in the DDR pathways (>100), and their contribution to PrCa risk has only more recently been explored. For example, two recent studies have provided evidence for a role of *CHEK2*, *PALB2*, *BRIP1*, *RAD51C*, *RAD51D*, *BARD1* and *NBN* in PrCa risk [18,19]. Importantly, tumours harbouring loss-of-function mutations in DDR genes exhibit a therapeutic response to poly (ADP-ribose) polymerase inhibitors (PARPi) [20] and platinum-based chemotherapy [21]. Thus, screening for clinically actionable germline variants in PrCa patients, particularly those with a significant family history together with advanced disease, represents an important strategy to improve PrCa outcomes. However, the rarity of these variants in population-based PrCa datasets, which represent the majority of PrCa DDR gene discovery studies to date, has hampered research advances. In addition, many of these studies have not differentiated between germline and acquired mutations, and those variants that have been identified remain largely of unknown clinical significance.

Curation of the full spectrum of DDR genetic variants contributing to PrCa risk has significant potential in the healthcare setting, where precision medicine can be implemented for both diagnosis and treatment. Moreover, the observation that germline and acquired mutations are frequently identified in the same DDR genes underscores the importance of these pathways in tumour development. Here, we interrogated whole-genome and -exome germline data from two high-risk familial PrCa datasets with the aim of identifying novel, rare DDR gene variants contributing to PrCa risk.

## 2. Methods

### 2.1. Study Resources

This study utilised clinicopathological and genetic data available from two independent PrCa resources: the Australian resource, consisting of the Tasmanian Familial Prostate Cancer Study and the population-based Tasmanian Prostate Cancer Case-Control Study, and the North American resource, consisting of the Prostate Cancer Genetic Research Study (*PROGRESS*) from the Fred Hutchinson Cancer Center (FHCC).

The Tasmanian Familial Prostate Cancer Study included 73 PrCa families comprising DNA from 379 affected men and 471 unaffected male and female relatives of Northern European heritage [22,23,24]. The study was initiated in Tasmania in the late 1990s, prior to the implementation of wide-spread prostate-specific antigen (PSA) testing as a PrCa screening tool. Families with more than two affected first-degree relatives spanning two or more generations were identified through interrogation of the Menzies Genealogical database and the Tasmanian Cancer Registry (TCR), in addition to collaboration with local clinicians.

The second, population-based Tasmanian Prostate Cancer Case-Control Study comprises 459 cases and 322 male controls of Northern European ancestry [22,23,24]. Cases were identified from the TCR. Controls were selected at random from the Tasmanian electoral roll, frequency matched by five-year age groups to the cases, and checked bi-annually against the TCR for PrCa diagnosis.

The FHCC resource comprises *PROGRESS*, which includes >300 PrCa families from across North America [25]. Whole exome sequencing (WES) data were available for 130 families, which included 11 older unaffected men and 321 affected men. Men prioritised for WES were those diagnosed with an early-onset/aggressive disease phenotype, uncle–nephew and/or cousin pairs from families with densely aggregated affected men.

Details of the clinical data available for these resources are presented in Table 1.

Additionally, age-at-diagnosis and Gleason score (GS) data from 2126 participants enrolled in the population-based Prostate Cancer Outcomes Registry—Tasmania (PCOR-TAS) were available for clinicopathological analyses. PCOR-TAS was established in 2015, with an aim to improve all aspects of the quality of care for men diagnosed with PrCa. The opt-out registry is an ongoing initiative that records data on the diagnosis, treatment, outcomes, and quality of life for Tasmanian men diagnosed with PrCa. Details of the registry, including data collection methods, have been described previously [26]. As of 15 July 2021, 2126 men had been recruited into PCOR-TAS, with ~3% having opted out of the registry.

### 2.2. Whole-Genome Sequencing and Bioinformatic Sequence Analysis

Whole-genome sequencing (WGS) data were generated from germline DNA (additional details: Supplementary Materials: Method S1) for 54 individuals from eight Australian families (Supplementary Materials: Table S1) and seven unaffected men from the Australian Case-Control Study.

Of the familial individuals, 43 had been diagnosed with PrCa, with the remaining individuals comprising a female relative with a self-reported breast cancer diagnosis (*n* = 1) and unaffected male relatives (*n* = 10). WGS (mean coverage = 38.7x; range = 29.2x–49.8x; the sequencing coverage and quality statistics for each sample are summarized in Supplementary Materials: Table S2) was completed in five instalments at the Australian Genome Research Facility (Melbourne, Australia), the Ramaciotti Centre for Genomics (Sydney, Australia), and the Texas Biomedical Research Institute (San Antonio, TX, USA). Sequence data were aligned to the hg19 reference genome with BWA-MEM [27], and variants were called with GATK [28], using bcbio-nextgen (https://github.com/bcbio/bcbio-nextgen, accessed on 27 June 2024).

### 2.3. Variant Filtering, Prioritisation, and Validation

A panel of 35 genes involved in DDR pathways was compiled (Supplementary Materials: Table S3), in addition to the established PrCa risk gene, *HOXB13* [29]. Variants located in a genomic window 1000 bp up and downstream of the nominated candidate genes were extracted from WGS data using bcftools [30] and annotated using ANNOVAR [31]. Included genes and genomic positions can be found in Supplementary Materials: Table S4.

**Figure 1:**
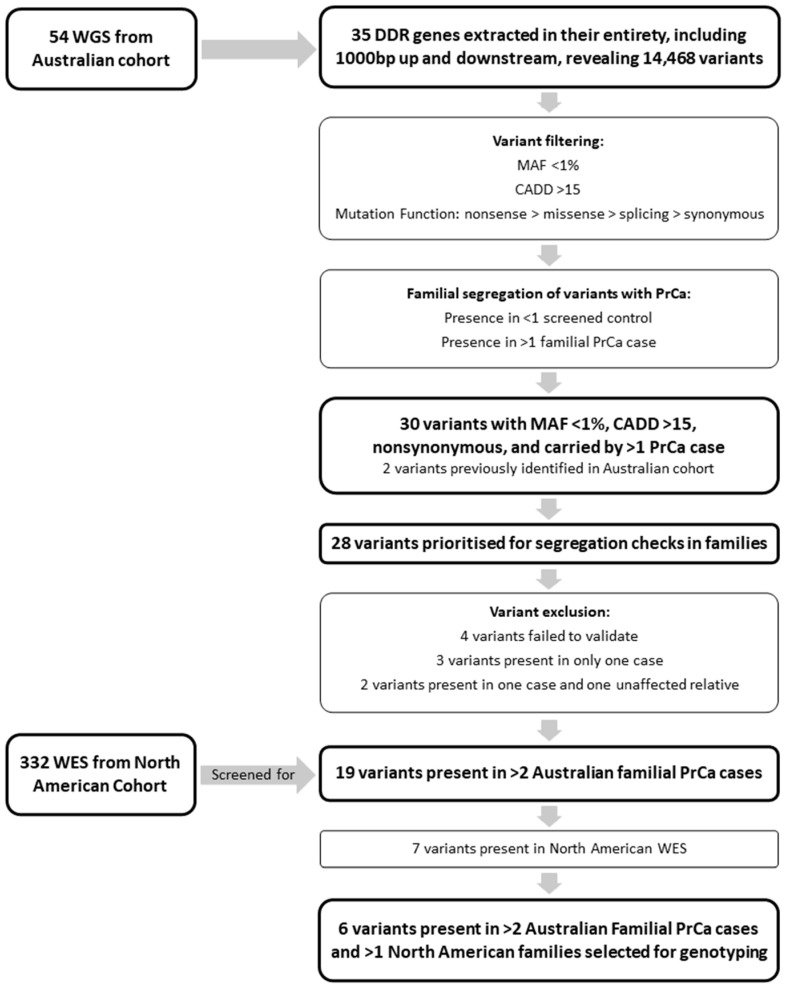
**Variant Filtering and Prioritisation Schematic**. Flow chart outlining genetic analysis pipeline including variant filtering and prioritisation of variants for follow-up.

Variant filtering and prioritisation occurred according to a range of criteria (Figure 1). Variants were filtered to include those with a minor allele frequency (MAF) < 1% in gnomAD non-Finnish Europeans (NFE) and Combined Annotation-Dependent Depletion (CADD) score > 15, with further prioritisation informed by predicted mutation function (e.g., nonsense > missense > splicing > synonymous). Variants were excluded if present in >1 of the seven screened unaffected male control genomes, or if present only in PrCa unaffected familial individuals.

Short-listed variants (MAF < 1%, CADD > 15, nonsynonymous, and carried by >1 PrCa case; (Figure 1)), which had been validated by Sanger sequencing on the ABI 3500 Genetic Analyser (Applied Biosystems, Foster City, CA, USA), were genotyped in additional non-WGS relatives to determine segregation in the relevant discovery family. Primers were designed to amplify fragments approximately 300 bp in length for each of the selected variants. Primer sequences are presented in Supplementary Materials: Table S5, and PCR conditions are available upon request.

### 2.4. Additional Genotyping in Expanded Australian Resources

Six prioritised variants were genotyped in the full Australian familial and case–control resources, using TaqMan™ genotyping assays (ThermoFisher Scientific, Waltham, MA, USA; Supplementary Materials: Table S6) on the LightCycler^®^ 480 system (Roche, Basel, Switzerland). Existing whole exome data were interrogated for prioritised gene variants in the *PROGRESS* study individuals.

### 2.5. Statistical Analysis

The distribution of clinical disease features in the Australian and *PROGRESS* prostate cancer cases is reported as percentages, median and interquartile range. Association between genotyped variants and PrCa risk was tested for using Modified Quasi-Likelihood Score (M_QLS_) analysis [32] (additional details: Supplementary Materials: Method S2) [33,34]. Population prevalence of PrCa was set at one in seven, and the analyses were conducted in the Australian familial and case–control datasets alone, the FHCC *PROGRESS* cohort alone, and all datasets combined. For DDR variants significantly associated with PrCa risk, a two-sided Wilcoxon rank-sum test was used to compare median diagnosis age of Tasmanian Familial, Sporadic and *PROGRESS* variant carriers with population-based PCOR-TAS cases (TAS) or the full *PROGRESS* cohort (FHCC). The distribution of variant carriers across several age-at-diagnosis categories (<50, <55, <60, <65, <70) is reported as numbers and percentages.

## 3. Results

### 3.1. Clinical Characteristics of Australian and North American PrCa Resources

Clinical characteristics of the study resources are presented in Table 1. Age-at-diagnosis, time interval between diagnosis and death, and proportion of PrCa-specific deaths were similar across the datasets.

### 3.2. Identification of Candidate Rare DDR PrCa Risk Variants

WGS data were interrogated for rare, potentially pathogenic variants in 35 DDR genes (Supplementary Materials: Table S3). Initial filtering identified 30 variants in 20 genes, of which two in *HOXB13* and *RAD51C* have previously been shown to be significantly associated with PrCa risk in our Australian cohort [22,23], providing proof-of-principle for our approach. Of the 28 remaining variants, four failed to validate via Sanger sequencing and were excluded from further investigation.

To determine segregation with disease, the remaining 24 variants underwent Sanger sequencing in additional non-WGS affected and unaffected relatives with DNA from each of the Australian discovery families (Table 2). Five variants were subsequently excluded: three variants that were each only present in a single affected man and two variants that were only present in a single affected man and one unaffected relative. The remaining 19 variants, *ATM* rs56128736, *BARD1* rs3738888, *BRCA1* rs28897673, *BRCA2* rs28897727, *BRCA2* rs55639415, *BRCA2* rs786202915, *BRIP1* rs4988345, *ERCC2* rs142568756, *ERCC3* rs145201970, *MRE11* rs777373591, *MSH6* rs142254875, *MUTYH* rs36053993, *PARP2* rs200603922, *PMS2* rs1554304601, *POLE* chr12: 133219216, *POLE* rs41561818, *PTEN* rs587779989, *PTEN* rs773513402, and *RECQL4* rs780723602, were present in at least two affected relatives from the Australian discovery cohort.

For further prioritisation, we then determined whether any of the 19 variants were present in the North American *PROGRESS* families. Examination of exome data from 332 individuals revealed seven variants in 34 cases from 22 kindreds. Four variants, *ATM* rs56128736, *BRCA2* rs28897727, *ERCC3* rs145201970, and *MUTYH* rs36053993, were present in two or more PrCa cases in a single family (Table 2).

Six DDR variants, *BARD1* rs3738888, *BRCA2* rs28897727, *BRIP1* rs4988345, *ERCC3* rs145201970, *MUTYH* rs36053993, and *PARP2* rs200603922, that partially segregated with disease in an Australian PrCa family and were present in two or more *PROGRESS* families, were selected for additional investigation (Table 3). These variants were genotyped in the extended Australian familial and case–control resources via TaqMan genotyping (Supplementary Materials: Table S6). All six variants were identified in additional individuals (n_range_ = 9 to 33; Supplementary Materials: Table S7) within the Australian datasets, and all except *MUTYH* rs36053993 were observed in additional familial PrCa cases. With the inclusion of the *PROGRESS* dataset, the *BARD1* rs3738888 and *BRIP1* rs4988345 variants were each observed in the most PrCa cases (n = 22), which included ten and nine sporadic cases, respectively. The predicted pathogenicity of these variants was determined using multiple bioinformatic tools (additional details: Supplementary Materials: Method S3) [35,36,37,38,39,40,41,42,43,44], and outputs are shown in Table 3.

### 3.3. Statistical Analysis

Genotypes were available for six variants in 1963 individuals, including 700 familial and 459 sporadic PrCa cases overall. M_QLS_ association analysis permitted the inclusion of related and unrelated individuals while also appropriately controlling for Type 1 error [32]. In the Australian dataset, a significant association was observed between *PARP2* rs200603922 and PrCa risk (*p* = 0.028), whilst in the *PROGRESS* dataset, a significant association was observed between *BRIP1* rs4988345 (*p* = 0.034), *ERCC3* rs145201970 (*p* = 0.010), and *MUTYH* rs36053993 (*p* = 0.031) and PrCa risk (Table 4). In the combined Australian and *PROGRESS* datasets, a significant association with PrCa risk was observed between *BRIP1* rs4988345 (*p* = 0.025) and *ERCC3* rs145201970 (*p* = 2.57 × 10^−4^). The *ERCC3* variant remained significant following Bonferroni correction for multiple testing. PrCa status of variant carriers is provided in Supplementary Materials: Table S7, and clinical characteristics of affected familial carriers are presented in Supplementary Materials: Table S8.

**Figure 2:**
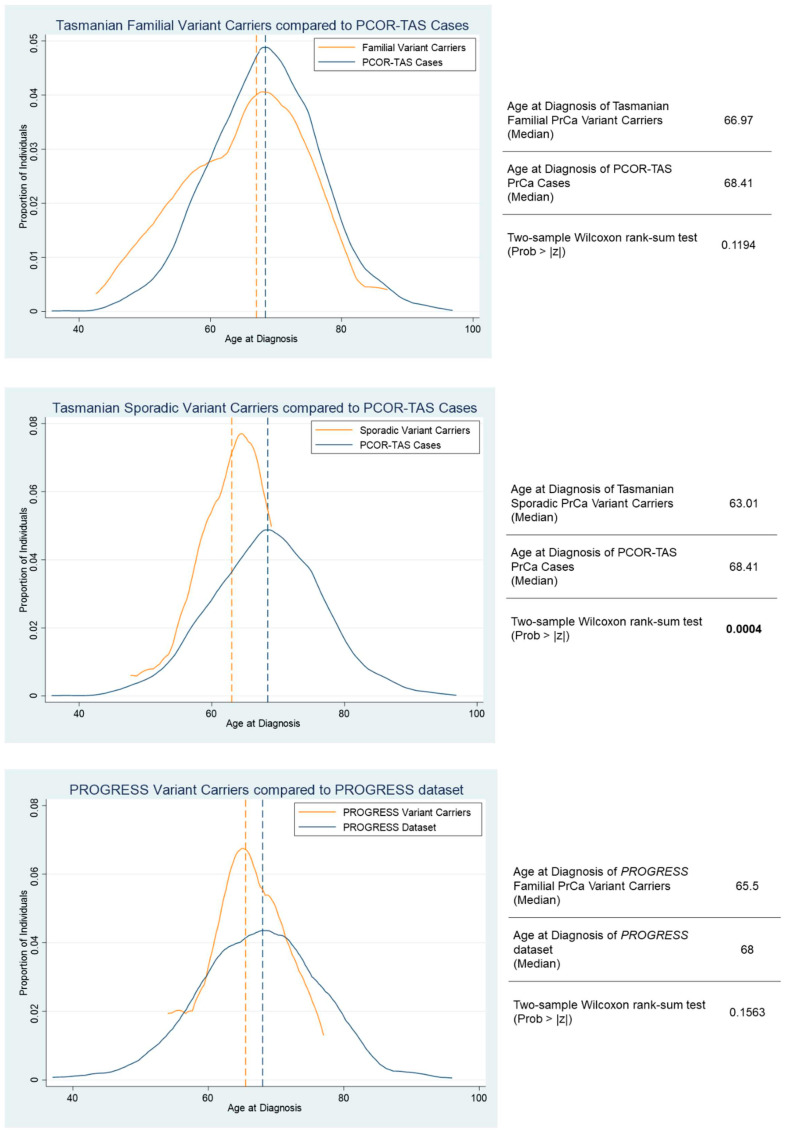
Age at diagnosis of DDR gene variant carriers* compared to available population-based datasets. Age at diagnosis of variant carriers compared with a comparable population dataset (presented as proportion of individuals versus age at diagnosis). Dashed vertical lines represent the median age of diagnosis. Top Panel: Familial PrCa variant carriers (n = 31) overlayed with unscreened PCOR-TAS case population (n = 2126); Middle Panel: Sporadic PrCa variant carriers (n = 22) compared with unscreened PCOR-Tas case population (n = 2126); and Bottom Panel: *PROGRESS* dataset variant carriers (n = 28) compared with non-variant carriers from the *PROGRESS* dataset. * DDR variant carriers = those carrying a pathogenic variant in *BRIP1*, *BRCA2, ERCC3*, *MUTYH*, *PARP2*, or *RAD51C*. Bolded text indicates significant results.

Age-at-diagnosis amongst variant carriers was compared to relevant population datasets (Figure 2), with a shift towards younger age-at-diagnosis observed, most evident in Australian sporadic rare variant carriers compared with the population-based PCOR-TAS cohort. While a slightly higher proportion of DDR variant carriers was observed in cases diagnosed before 55 years of age (9.4%; Supplementary Materials: Table S9), variant carriers were relatively consistent at ~6% across the remaining age-at-diagnosis categories. Population data from PCOR-TAS revealed that ~20% of Tasmanian men were diagnosed with a GS ≥ 8. Of men carrying a risk DDR variant, 23% (15/66) were diagnosed with GS ≥ 8, while 58% (38/66) of rare variant carriers were diagnosed with a GS ≤ 6.

## 4. Discussion

The discovery of rare, high-risk germline variants has long proven challenging due to their very low frequency, which substantially impacts power to detect significant statistical associations. However, there remains considerable impetus to characterise rare risk variants in DDR genes, especially considering the increasing availability of therapies targeting this pathway. In a candidate gene approach designed to take advantage of large familial PrCa resources, where rare risk variants are expected to be enriched, we examined massively parallel sequencing data from two independent datasets to identify rare, likely deleterious DDR variants. Subsequent analysis of 1963 individuals from the Australian and *PROGRESS* datasets revealed statistically significant associations between rare variants in *ERCC3* and *BRIP1* and PrCa risk, with *ERCC3* surviving correction for multiple testing. In addition, a variant in *PARP2* was significantly associated with PrCa risk in the Australian dataset alone, while a variant in *MUTYH* was significantly associated with PrCa risk only in the *PROGRESS* dataset.

*ERCC3* encodes one of two ATP-dependent DNA helicases, which are core members of the nucleotide excision repair pathway. The *ERCC3* rs145201970 variant (MAF 0.17%), located in exon 7, causes an amino acid change at position 283 (p.R283C), which is predicted to disrupt the arginine-aspartic acid salt bridge via the inclusion of a more hydrophobic residue. The variant is located within two domains listed by Interpro as likely required for ERCC3 protein function [45]. Topka et al. comprehensively examined germline mutations in the *ERCC2*, *3*, *4,* and *5* genes in 16,712 patients affected by multiple different cancers [46]. Numerous likely pathogenic/pathogenic loss of function (LoF) germline variants were observed in *ERCC3*, with rs145201970 (n = 42) representing the second most observed LoF variant in this gene in cancer patients after rs34295337 (n = 70) [46]. While there are no previous reports describing rs145201970 as a PrCa risk variant, other germline pathogenic/likely pathogenic *ERCC3* variants in PrCa patients have been recently reported by Kohaar et al. [47], Carignan et al. [48] and Rantapero et al. [49]. Additionally, an intronic *ERCC3* variant has been associated with increased risk of biochemical recurrence after low-dose-rate prostate brachytherapy, potentially due to reduced mRNA expression in variant carriers [48]. In breast cancer, a recurrent truncating mutation has been associated with familial disease [50,51]. In vitro studies have demonstrated that mutations in *ERCC3* impair DNA repair capability and confer a selective sensitivity to Irofulven, a sesquiterpene that has demonstrated some efficacy in clinical trials for metastatic PrCa [46].

*BRIP1* is a member of the Fanconi Anaemia gene family and functions in the double-strand break repair pathway, interacting closely with *BRCA1*. The rare rs4988345 variant (MAF 0.43%) is in exon 5, located within the nuclear localisation signal domain. As a result of the p.R173C amino acid change, there is a loss of positive charge and a more hydrophobic residue introduced within a helicase ATP-binding domain and a region annotated as a nuclear localization signal. *BRIP1* rs4988345 has been previously identified in a study enriched for familial PrCa but was only observed in a single PrCa case (0.52%) [52]. Other rare *BRIP1* variants were detected in five hereditary PrCa cases (MAF < 1%) [53]; however, no statistical analyses were performed due to their low frequency. *BRIP1* has been included on screening panels for several clinical trials investigating the response of metastatic PrCa patients with DDR defects to Olaparib, a PARPi (ClinicalTrials.gov Identifier: NCT02987543) [54]. A cohort of that study comprised men harbouring mutations in 12 DDR genes, including *BRIP1,* however, only four individuals were identified as carriers of a variant in this gene, below the pre-set threshold for statistical analysis. Evaluation of *BRIP1* has also been included in the Phase 2 TRITON2 trial (ClinicalTrials.gov Identifier: NCT02952534), where one patient with a *BRIP1* variant responded to the PARPi, Rucaparib [55].

*PARP2* is a poly (ADP-ribose) polymerase involved in the base excision repair pathway (BER), and rs200603922 is located in the first exon (p.R15G). This variant (MAF 0.12%) has previously been observed to partially segregate with PrCa in familial cases who tested negative for *BRCA1* and *BRCA2* mutations [56]. Although several bioinformatic tools predict the variant allele to be benign (Table 3), the R15G amino acid change introduces a more hydrophobic residue, which may impact protein interactions and the phosphorylation of distal residues. There is one other report of a *PARP2* variant, rs3093926 (MAF 4.2%), segregating in a PrCa pedigree, but the contribution of this variant to PrCa risk remains undetermined [57], and though common, it was not observed in our Australian discovery families. *PARP2* mutations have been associated with breast cancer risk [58], but similarly to PrCa, their functional impact remains unclear. However, *PARP2* remains of interest given the ongoing development of PARPi. Though most primarily target *PARP1*, some, such as Niraparib [59], also affect *PARP2,* which may be relevant when assessing therapeutic PARPi in men with *PARP2* mutations.

*MUTYH* encodes a DNA glycosylase involved in oxidative DDR and the BER pathway. The rs36053993 variant (MAF 0.47%) results in an amino acid change from a neutral residue to a negatively charged, less hydrophobic residue (p.G368D), with this change located in the highly conserved nudix hydrolase domain. The NCBI human variant database, ClinVar, lists this variant as pathogenic/likely pathogenic arising from its association with MUTYH-associated polyposis, an autosomal recessive hereditary condition typified by the development of colorectal carcinomas. Kohaar et al. (2022) previously reported the rs36053993 SNP in germline samples from PrCa patients [47]. Others have also reported several pathogenic/likely pathogenic variants in *MUTY*H, including a study reporting 1.8% of 1351 PrCa cases [60] and another reporting 2.4% of 3607 PrCa cases as carrying pathogenic variants in this gene [61]. Furthermore, reduced gene and protein expression of *MUTYH* in prostate tumours has been associated with an increase in total somatic mutations, which may result from impaired DDR capacity [62].

In this study, the strategy for filtering and prioritisation of variants was developed to detect moderate to highly penetrant, rare DDR gene variants that contribute to familial PrCa risk. It is notable that rare germline variants predicted to be deleterious have been previously observed in *BRIP1* (n = 7), *ERCC3* (n = 8), *MUTYH* (n = 10), and *PARP2* (n = 5) in a cohort of 5545 non-aggressive and aggressive sporadic PrCa cases [12]. While no statistically significant association with aggressive disease risk was observed, due to the very low frequency of these variants, their association with PrCa risk in general was not explored in this case-only cohort (see Supplementary Tables, Darst et al. [12]). Notably, this study examined 5545 cases, and statistically significant associations with aggressive disease were only demonstrated for previously known PrCa risk genes, *BRCA2* and *PALB2*, with a nominal association seen for *ATM*. This highlights the fact that the innate rarity of DDR gene variants presents a significant challenge for rare variant evaluation, even in larger sporadic case datasets. Our approach was designed to maximise power by seeking to identify rare variants enriched in two large familial PrCa cohorts. However, it is possible that additional rare, disease-associated variants were not detected due to not being present in the Australian WGS discovery cases. It was also necessary to restrict follow-up to only those candidate variants observed in more than one North American family, as the rareness of these variants impacts statistical power to detect associations. However, this may have resulted in risk variants associated with disease in the Australian cohort being missed, e.g., *ATM* rs56128736. Furthermore, instances where prioritised variants were subsequently not found to be associated with PrCa could be attributed to their rarity and, thus, lack of statistical power. Thus, to establish the necessary evidence base to inform clinical decision making, it is critical that both our significant DDR risk variants and all prioritised variants be validated/examined in additional familial and population-based datasets, including large publicly available resources such as the UK Biobank. Concurrently, it is worth considering the expansion of candidate gene screening strategies in current clinical trials of PARPi and in current germline testing guidelines for men with a family history of PrCa.

Examining rare variant association with clinicopathological variables presented significant challenges, again due to their rarity, but also due to biases introduced by recruitment strategies. For example, while the aim was to collect all known relatives with PrCa in the Australian and *PROGRESS* familial cohorts, population-based cases with an early age-at-diagnosis were targeted for recruitment to the Australian case–control study.

Carriers of putative pathogenic DDR variants were slightly more frequently observed in the earlier age-at-diagnosis group, and mildly increased in the GS ≥ 8-at-diagnosis group when compared with the population-based PCOR-TAS cohort. However, it is notable that the majority of variant carriers were diagnosed with a GS ≤ 6 (58%) and/or at age ≥65 years (53%), consistent with the findings of Darst et al. [12], where carriers of DNA repair mutations were, on average, diagnosed only ~1 year younger than non-carriers. Taken together, these findings raise the question as to whether limiting screening for putative genetic DDR variants to very early-onset (<50 years) or only high-grade disease patients is likely to result in a substantial proportion of DDR variant carriers being excluded from testing, and subsequently denied access to effective treatment modalities.

## 5. Conclusions

This study implicates several additional DDR genes as contributors to inherited genetic risk of PrCa. The existing evidence that rare DDR gene variants are associated with aggressive disease and the growing use of cancer therapies targeting this pathway highlights the potential significance of these findings. However, this study raises the concern that confining genetic screening to only those patients with early-onset and/or high-grade PrCa may result in a missed opportunity for some men to receive effective, targeted gene-based therapies.

## Figures and Tables

**Table 1:** Clinical characteristics of study resources with available genetic data.

	Australian Familial Cases, *n* (%)	Australian Sporadic Cases, *n* (%)	*PROGRESS* Familial Cases, *n* (%)
Age at Diagnosis			
<60	93 (24.54%)	137 (29.85%)	108 (33.64%)
60–64	98 (25.86%)	170 (37.04%)	75 (23.36%)
65–69	97 (20.32%)	125 (27.23%)	77 (23.99%)
≥70	107 (28.23%)	26 (5.66%)	61 (19.00%)
Missing	4 (1.06%)	1 (0.22%)	n.a.
Age at Diagnosis, Median (IQR)	64.82 (60.06–71.50)	62.59 (59.26–66.05)	63 (57.0–68.0)
Years between Diagnosis and Death			
<5	19 (5.01%)	25 (5.45%)	11 (3.43%)
5–9	39 (10.29%)	54 (11.76%)	36 (11.21%)
10–19	81 (21.37%)	103 (22.44%)	65 (20.25%)
≥20	18 (4.75%)	19 (4.14%)	7 (2.18%)
Missing	2 (0.53%)	n.a.	n.a.
n.a.	220 (58.05%)	258 (56.21%)	202 (62.93%)
Years between Diagnosis and Death, Median (IQR)	11.62 (8.75–16.10)	11.82 (7.64–15.52)	11.00 (6.50–15.0)
Cause of Death			
PrCa	49 (12.93%)	68 (14.81%)	41 (12.77%)
Other	91 (24.01%)	133 (28.98%)	72 (22.43%)
Not Processed	2 (0.53%)	n.a.	n.a.
Missing	17 (4.49%)	1 (0.22%)	6 (1.87%)
n.a.	220 (58.05%)	257 (55.99%)	202 (62.93%)
Total	379	459	321

IQR = Interquartile Range; n.a. = Not Applicable; PrCa = Prostate Cancer.

**Table 2:** Putative pathogenic mutations identified in DDR genes in Australian discovery and North American families.

Gene	Variant	Amino Acid Change	MAF (gnomAD NFE) *	Australian Discovery Familial Cohort			North American Familial Cohort		
				PrCa Affected Carriers	Total Carriers	Number of Families	PrCa Affected Carriers	Total Carriers	Number of Families
*ATM*	rs55801750	C2464R	9 × 10^−4^	1	2	1	-	-	-
*ATM*	rs55982963	R2719H	1 × 10^−4^	1	2	1	-	-	-
*ATM*	rs56128736	V410A	0.0021	3	6	2	2	2	1
*ATM*	rs767507047	Y2954C	6.48 × 10^−5^	1	1	1	-	-	-
*BRCA1*	rs28897673	Y58C	1 × 10^−4^	2	4	1	-	-	-
*BRCA2*	rs55639415	S1733F	4.71 × 10^−5^	2	4	1	-	-	-
*BRCA2*	rs56403624	E462G	4.29 × 10^−4^	1	1	1	-	-	-
*BRCA2*	rs786202915	F2254Y	n.a.	3	8	1	-	-	-
*ERCC2*	rs142568756	V536M	0.0005	2	4	1	-	-	-
*MRE11*	rs777373591	P132S	1.77 × 10^−5^	2	4	1	-	-	-
*MSH6*	rs142254875	P943S	0.0001	2	6	1	-	-	-
*PMS2*	rs1554304601	A116V	n.a.	3	7	1	-	-	-
*POLE*	chr12: 133219216	P1610A	n.a.	3	9	1	-	-	-
*POLE*	rs36120395	P697R	0.0016	1	1	1	-	-	-
*POLE*	rs41561818	A1420V	0.0044	2	3	1	-	-	-
*PTEN*	rs587779989	n.a.	n.a.	3	6	1	-	-	-
*PTEN*	rs773513402	n.a.	0.0003	2	5	1	-	-	-
*RECQL4*	rs780723602	I920V	9 × 10^−6^	4	11	1	-	-	-
*BARD1*	rs3738888	R658C	0.0063	2	5	1	4	4	4
*BRCA2*	rs28897727	D1420Y	0.0098	3	9	1	4	5	3
*BRIP1*	rs4988345	R173C	0.0043	2	5	1	6	6	6
*ERCC3*	rs145201970	R283C	0.0017	2	7	1	5	5	3
*MUTYH*	rs36053993	G368D	0.0047	2	5	2	9	9	4
*PARP2*	rs200603922	R15G	0.0012	4	6	1	2	2	2

n.a. = Not Applicable. * MAF from gnomAD version 4.

**Table 3:** Predicted pathogenicity of prioritised DDR gene variants.

Gene	Variant	Chr: Position °	Allele Change	AA Change	CADD *	DANN **	SIFT	PROVEAN	PolyPhen	Mutation Taster (Rank Score)	Mutation Assessor	FATHMM † (Coding)
*BARD1*	rs3738888	2:214730440	G > A	R658C	24.3	0.999	0.008 (D)	−4.02 (De)	1 (P)	0.462 (D)	2.12 (M)	0.9778
*BRCA2*	rs28897727	13:32338613	G > T	D1420Y	16.66	0.988	0.030 (D)	−6.60 (De)	0.03 (B)	0.09 (N)	2.15 (M)	0.49798
*BRIP1*	rs4988345	17:61847211	G > A	R173C	24.7	0.999	0.001 (D)	−2.54 (De)	1 (P)	0.81 (D)	2.67 (M)	0.93639
*ERCC3*	rs145201970	2:127288840	G > A	R283C	26.5	0.999	0.000 (D)	−7.59 (De)	0.995 (P)	0.81 (D)	3.31 (M)	0.99364
*MUTYH*	rs36053993	1:45331556	C > T	G368D	29.7	0.998	0.000 (D)	−6.46 (De)	1 (P)	0.81 (D)	4.09 (H)	0.99757
*PARP2*	rs200603922	14:20343684	A > G	R15G	16.46	0.8	0.153 (T)	−1.04 (N)	0 (B)	0.09 (N)	0.695 (N)	0.00048

D = Damaging; T = Tolerated; De = Deleterious; N = Neutral; P = Probably Damaging; B = Benign; M = Medium; H = High. ° Hg38 dbSNP Release 155; * CADD GRCh38-v1.6; ** DANN predictions use a scoring system between 0 and 1, with scores closer to one indicating greater predicted pathogenicity; † FATHMM predictions use a scoring system between 0 and 1, with scores closer to one indicating greater predicted pathogenicity.

**Table 4:** Carrier frequency and statistical analysis of variants.

Gene	Variant	Australian Familial and Sporadic Prostate Cancer			North American Familial *PROGRESS* Cohort			Tasmanian Familial Prostate Cancer Study and *PROGRESS*		
		Total Carriers (% Cases) *	M_QLS_ *p*-Value	M_QLS_ Odds Ratio	Total Carriers (% Cases) *	M_QLS_ *p*-Value	M_QLS_ Odds Ratio	Total Carriers (% Cases) *	M_QLS_ *p*-Value	M_QLS_ Odds Ratio
*BARD1*	rs3738888	31 (58.1%)	0.407	1.7	4 (100%)	0.318	n.a.	35 (62.9%)	0.066	1.9
*BRCA2*	rs28897727	24 (54.2%)	0.063	n.a.	5 (80%)	0.157	n.a.	29 (58.6%)	0.193	n.a.
*BRIP1*	rs4988345	25 (64.0%)	0.118	3.1	6 (100%)	**0.034**	**n.a.**	31 (71.0%)	**0.025**	**3.1**
*ERCC3*	rs145201970	16 (50.0%)	0.554	1	5 (100%)	**0.010**	**n.a.**	21 (61.9%)	**2.57 × 10^−4^**	**1.7**
*MUTYH*	rs36053993	23 (26.1%)	0.630	0.4	9 (100%)	**0.031**	**n.a.**	32 (46.9%)	0.201	0.8
*PARP2*	rs200603922	14 (71.4%)	**0.028**	**n.a.**	2 (100%)	0.388	n.a.	16 (75.0%)	0.162	n.a.

n.a. = Not Applicable, as the odds ratio cannot be calculated when no carriers in controls are identified. * % of variant carriers that are cases. Bolded text indicates significant results.

## Data Availability

The data that support the findings of this study are available from the corresponding author upon reasonable request. The genome and exome sequencing data are not publicly available due to privacy or ethical restrictions.

## Supplementary Materials

The following supporting information can be downloaded at: https://www.mdpi.com/article/10.3390/cancers16132482/s1. Method S1: Nucleic Acid Extractions and Whole Genome Sequencing; Method S2: Statistical Association Analysis; Method S3: Bioinformatic Predictions; Table S1: Summary of individuals from the Australian discovery cohort with WGS data; Table S2: Sequencing coverage and quality assessment of each sample with massively parallel sequencing data; Table S3: DNA repair pathway genes included for analysis; Table S4: Genes extracted from WGS data; Table S5: Primers used for Sanger sequencing; Table S6: TaqMan™ genotyping assay information; Table S7: Prostate cancer status of variant carriers; Table S8: Clinical characteristics of affected familial DDR variant carriers; Table S9: DDR variant rates by age at diagnosis. Refs. [33,34,35,36,37,38,39,40,41,42,43,44] are cited in Supplementary Materials.
