# Supplementary material for: Germline Sequencing of DNA Damage Repair Genes in Two Hereditary Prostate Cancer Cohorts Reveals New Disease Risk-Associated Gene Variants
Source: Cancers (Basel). 2024 Jul 7;16(13):2482. doi: 10.3390/cancers16132482 (PMC11240467; doi:10.3390/cancers16132482)
Supplement: Supplementary file 1 [file cancers-16-02482-s001.zip › cancers-3049951-supplementary.pdf]

SUPPLEMENTARY METHODS

Supplementary Method S1: Nucleic Acid Extractions and Whole Genome Sequencing

Germline DNA was extracted from either 10 mL of peripheral blood using the Nucleon BACC3 Kit (GE Healthcare) or from 4 mL of saliva using the Oragene OG-500 DNA collection tubes (DNA Genotek), following the manufacturers' directions. All DNA samples were quantified using a Nanodrop 8000 UV-vis spectrophotometer (Thermofisher Scientific) and normalised for whole-genome sequencing (WGS) and/or TaqMan™ genotyping.

Supplementary Method S2: Statistical Association Analysis

Modified Quasi-Likelihood Score (M_{QLS}) analysis permits the combined analysis of genotype data from both familial and case–control samples, taking relatedness into consideration. It also distinguishes between unaffected and uncertain phenotypes, incorporating both into the analyses. M_{QLS} uses variance components to examine the significance of association for related individuals, and when the disease status is known for first-degree relatives of cases, M_{QLS} obtains additional power by giving increased weighting to those individuals with closely related disease-carrying relatives. The use of this approach maximises power but does not inflate type 1 error [1, 2].

Supplementary Method S3: Bioinformatic Predictions

The predicted structural and functional effects of candidate risk variants was evaluated using the online analysis server, HOPE [3]. Protein sequences and amino acid information were sourced via the UCSC genome browser [4]. Additional bioinformatic tools, including DANN [5], Sift [6], PROVEAN [7], PolyPhen [8], Mutation Taster [9], Mutation Assessor [10], FATHMM [11], and Protein Predict [12], were used to appraise the predicted pathogenicity of candidate variants.

References

1. Hong, X.; Hao, K.; Ladd-Acosta, C.; Hansen, K.D.; Tsai, H.-J.; Liu, X.; Xu, X.; Thornton, T.A.; Caruso, D.; Keet, C.A.; et al. Genome-wide association study identifies peanut allergy-specific loci and evidence of epigenetic mediation in US children. *Nat. Commun.* **2015**, *6*, 6304, doi:10.1038/ncomms7304.
2. Cummings, A.C.; Torstenson, E.; Davis, M.F.; D'aoust, L.N.; Scott, W.K.; Pericak-Vance, M.A.; Bush, W.S.; Haines, J.L. Evaluating Power and Type 1 Error in Large Pedigree Analyses of Binary Traits. *PLOS ONE* **2013**, *8*, e62615, https://doi.org/10.1371/journal.pone.0062615.
3. Venselaar, H.; Beek, T.A.T.; Kuipers, R.K.; Hekkelman, M.L.; Vriend, G. Protein structure analysis of mutations causing inheritable diseases. An e-Science approach with life scientist friendly interfaces. *BMC Bioinform.* **2010**, *11*, 548–548, https://doi.org/10.1186/1471-2105-11-548.
4. Kent, W.J.; Sugnet, C.W.; Furey, T.S.; Roskin, K.M.; Pringle, T.H.; Zahler, A.M.; Haussler, D. The Human Genome Browser at UCSC. Genome Res. 2002, 12, 996–1006, doi:10.1101/gr.229102.
5. Quang, D.; Chen, Y.; Xie, X. DANN: a deep learning approach for annotating the pathogenicity of genetic variants. *Bioinformatics* **2014**, *31*, 761–763, https://doi.org/10.1093/bioinformatics/btu703.
6. Ng, P.C.; Henikoff, S. SIFT: predicting amino acid changes that affect protein function. *Nucleic Acids Res.* **2003**, *31*, 3812–3814, https://doi.org/10.1093/nar/gkg509.
7. Choi, Y.; Sims, G.E.; Murphy, S.; Miller, J.R.; Chan, A.P. Predicting the Functional Effect of Amino Acid Substitutions and Indels. *PLoS ONE* **2012**, *7*, e46688. https://doi.org/10.1371/journal.pone.0046688.
8. A Adzhubei, I.; Schmidt, S.; Peshkin, L.; E Ramensky, V.; Gerasimova, A.; Bork, P.; Kondrashov, A.S.; Sunyaev, S.R. A method and server for predicting damaging missense mutations. *Nat. Methods* **2010**, *7*, 248–249, https://doi.org/10.1038/nmeth0410-248.
9. Schwarz, J.M.; Cooper, D.N.; Schuelke, M.; Seelow, D. MutationTaster2: mutation prediction for the deep-sequencing age. *Nat. Methods* **2014**, *11*, 361–362. https://doi.org/10.1038/nmeth.2890.
10. Reva, B.; Antipin, Y.; Sander, C. Predicting the functional impact of protein mutations: application to cancer genomics. *Nucleic Acids Res.* **2011**, *39*, e118–e118, https://doi.org/10.1093/nar/gkr407.
11. A Shihab, H.; Gough, J.; Mort, M.; Cooper, D.N.; Day, I.N.; Gaunt, T.R. Ranking non-synonymous single nucleotide polymorphisms based on disease concepts. *Hum. Genom.* **2014**, *8*, 11–11, https://doi.org/10.1186/1479-7364-8-11.
12. Bernhofer, M.; Dallago, C.; Karl, T.; Satagopam, V.; Heinzinger, M.; Littmann, M.; Olenyi, T.; Qiu, J.; Schütze, K.; Yachdav, G.; et al. PredictProtein - Predicting Protein Structure and Function for 29 Years. *Nucleic Acids Res.* **2021**, *49*, W535–W540, https://doi.org/10.1093/nar/gkab354.

SUPPLEMENTARY TABLES

Supplementary Table S1: Summary of individuals from the Australian discovery cohort with WGS data

Family	PrCa Cases	PrCa Unaffected Relatives	Total
PC2	7	2	9
PC3	5	0	5
PC4	5	0	5
PC9	8	1	9
PC12	2	1	3
PC18	3	0	3
PC22	9	3	12
PC72	4	4	8
Total	43	11	54

Supplementary Table S2. Sequencing coverage and quality assessment of each sample with massively parallel sequencing data

Sample ID	Total Number of Sequenced Reads	Total Number of Uniquely Mapped Non-Duplicate Reads	Total Number of Covered Bases*	Median Coverage Per Targeted Base*	Percentage of Targeted Bases* with Coverage >10
**PC12-1**	862,696,604	609940546	2862935594	29.2 X	91.6038652
**PC12-132**	843,440,964	681552067	2863120241	32.1 X	91.90108053
**PC12-96**	818,961,420	634154539	2863063529	29.4 X	91.73530133
**PC18-1**	843,794,214	730022221	2863024724	35.7 X	92.02134509
**PC18-26**	1,065,066,542	893765031	2863146350	43.4 X	92.13105074
**PC18-57**	814,548,222	703094913	2862996990	34.3 X	91.96079534
**PC2-1**	1,287,737,198	1208282388	2863241969	40.3 X	92.06690499
**PC2-13**	866,718,652	737844504	2863008492	36.5 X	91.97758934
**PC2-16**	803,544,048	697843076	2862959273	34.3 X	91.91910761
**PC2-2**	1,315,119,794	1238705820	2863444380	41.3 X	92.09313498
**PC2-21**	750,826,484	651391649	2863129261	31.9 X	91.83045139
**PC2-25**	750,496,998	648604740	2842129530	31.4 X	91.3303506
**PC2-27**	824,304,972	710171178	2862839688	35.1 X	91.94756392
**PC2-3**	1,201,509,970	1131759098	2863351060	37.8 X	92.01792753
**PC2-48**	690,302,138	602347842	2862714494	29.2 X	91.58962482
**PC209-58**	1,009,250,358	885538110	2862925098	44.0 X	92.11139947
**PC22-02**	1,009,950,748	757531906	2863415922	36.1 X	92.08898852
**PC22-03**	1,472,419,426	1376148708	2863348265	45.8 X	92.15041002
**PC22-04**	1,257,790,960	1186554661	2863370280	39.8 X	92.07078947
**PC22-16**	1,321,798,116	1234857090	2863378526	41.1 X	92.07443074
**PC22-162**	1,371,351,236	1288533453	2863289418	42.5 X	92.09580207
**PC22-17**	1,450,003,614	1371849538	2863540998	45.8 X	92.15750325
**PC22-203**	1,375,907,510	1302884195	2863333543	43.3 X	92.12540787
**PC22-21**	1,124,165,512	1061057059	2863401506	35.2 X	91.95214957
**PC22-274**	1,014,045,430	962131962	2863413350	32.0 X	91.81089255
**PC22-387**	1,457,992,794	1379229418	2863506203	45.7 X	92.15752127
**PC22-388**	1,430,268,186	1346653896	2863494490	44.4 X	92.15601959
**PC22-584**	819,333,892	662043699	2862952258	30.7 X	91.8061938
**PC3-1**	930,488,034	687784958	2862999105	33.3 X	91.95877249
**PC3-2**	923,469,922	620417337	2863124933	30.1 X	91.764744
**PC3-31**	839,106,934	719924209	2863021783	33.6 X	91.97699024
**PC3-44**	962,674,914	667629567	2863180761	31.7 X	91.90265387
**PC3-8**	809,544,552	636624991	2863088132	29.6 X	91.80150721

**PC4-1**	851,603,418	688267461	2862936158	32.1 X	91.90339586
**PC4-161**	985,136,988	883172210	2862923913	43.7 X	92.11717914
**PC4-2**	961,987,064	848437248	2862881822	42.0 X	92.06348392
**PC4-3**	945,026,756	851651678	2862616252	42.0 X	92.07713403
**PC4-95**	1,028,704,450	893879175	2863186071	44.0 X	92.14500755
**PC72-02**	1,557,010,276	1474289422	2863525769	49.3 X	92.20634462
**PC72-03**	1,445,307,944	1356287997	2863382227	45.1 X	92.18005515
**PC72-04**	1,520,462,984	1399084411	2863307273	46.1 X	92.12724306
**PC72-106**	1,317,419,358	1243890365	2863176992	41.4 X	92.07940091
**PC72-126**	1,551,750,336	1449649328	2863439686	48.7 X	92.18702019
**PC72-188**	1,339,316,404	1263748018	2863344252	42.0 X	92.09831686
**PC72-75** **WES**	79,216,014	71363059	1228897543	5.1 X	3.201359188
**PC72-94**	1,524,604,004	1404306540	2863668051	47.0 X	92.20813606
**PC72-97**	1,585,587,164	1491980697	2844358112	49.8 X	91.51868163
**PC9-1**	901,562,094	752048541	2862838150	36.5 X	92.05677012
**PC9-126**	883,818,304	750928477	2862893783	36.2 X	92.04088198
**PC9-158**	1,044,246,234	863145074	2862926443	41.8 X	92.13332422
**PC9-3**	831,176,716	714615677	2862820304	35.3 X	91.98320178
**PC9-338**	772,808,108	665645424	2862781711	32.4 X	91.86365928
**PC9-477**	874,652,362	738818115	2863011420	36.2 X	92.02776081
**PC9-5**	889,864,306	762052443	2862526932	36.6 X	92.02169679
**PC9-588**	1,043,203,776	874456110	2863131767	42.6 X	92.14258977
**PC9-595**	937,083,482	788785312	2862874288	38.4 X	92.08953862

Supplementary Table S3: DNA repair pathway genes included for analysis

Pathway	Number of Genes	Gene List
Base Excision Repair	2	*MUTYH, PARP2*
Cell Cycle Regulation	5	*CDH1, CDKN1B, CDKN2Z, PTEN, STK11*
DNA Damage Response	3	*ATM, CHEK2, TP53*
Fanconi Anaemia	5	*BRCA2, BRIP1, PALB2, RAD51C, SLX4*
Homologous Recombination	9	*BARD1, BLM, BRCA1, GEN1, MRE11A, NBN, RAD50, RAD51D, RECQL4*
Mismatch Repair	7	*MLH1, MSH2, MSH5, MSH6, PMS2, POLD1, POLE*
Nucleotide Excision Repair	3	*ERCC2, ERCC3, XPC*
Included as Proof-of-principle	1	*HOXB13*
Total	36	

Supplementary Table S4: Genes extracted from WGS data

Gene	Chromosome	Start Position (bp*)	End Position (bp*)
ATM	11	108092559	108240826
BARD1	2	215592275	215675428
BLM	15	91259579	91359686
BRCA1	17	41195312	41278500
BRCA2	13	32888617	32974809
BRIP1	17	59755547	59941920
CDH1	16	68770195	68870444
CDKN1B	12	12869302	12876305
CDKN2A	9	21966751	21976132
CHEK2	22	29082731	29138822
ERCC2	19	45853649	45874845
ERCC3	2	128013866	128052752
GEN1	2	17934177	17967632
HOXB13	17	46801127	46807111
MLH1	3	37033841	37093337
MRE11A	11	94149469	94228040
MSH2	2	47629206	47711367
MSH5	6	31706725	31731945
MSH6	2	48009221	48033092
MUTYH	1	45793914	45807142
NBN	8	90944564	90997899
PALB2	16	23613483	23653678
PARP2	14	20810773	20827063
PMS2	7	6011870	6049737
POLD1	19	50886580	50922275
POLE	12	133199348	133264945
PTEN	10	89622195	89729532
RAD50	5	131891616	131981313
RAD51C	17	56768963	56812692
RAD51D	17	33425811	33434500
RECQL4	8	145735667	145744210
SLX4	16	3630184	3662585
STK11	19	1204798	1229434
TP53	17	7570720	7591868
XPC	3	14185648	14221172

*Gene positions according to hg19.

Supplementary Table S5: Primers used for Sanger sequencing

Gene	Variant	Forward Primer (5'–3')	Reverse Primer (5'–3')	Amplicon Size (bp)
ATM	rs55801750	AAAGGTTCAGCGAGAGCTGG	GATTCCTGACATCAAGGGGCT	273
ATM	rs55982963	TCTAAATGAAAGAATGGCAGTAGGT	CCTAGTTTCCGTGTTTCTCTGC	250
ATM	rs767507047	GATGTTTGTTCCCCTCCCCC	TGAAAAACTGACAACAGGACCTT	305
ATM	rs56128736	TGAAGATACCAGATCCTTGGAGA	AGGTTTGGGGGTAGACAAATGA	285
BARD1	rs3738888	AGCTGTTGAAAGGGCAGAAGT	TGCCATGAAGAAGAAAAACCACT	293
BRCA1	rs28897673	ACCAAGGAAGGATTTTCGGGT	CACAACAAAGAGCATACATAGGGT	243
BRCA2	rs786202915	TTCTGATGTTCCTGTGAAAACAAA	GTGATTGGCAACACGAAAGGT	279
BRCA2	rs55639415	ACTTCTGTGAGTCAGACTTCATT	TCTTCAATACTGGCTCAATACCAG	298
BRCA2	rs56403624	GTACCGTCTTTGGCCTGTGA	TTGCCTGCTTTACTGCAAGA	301
BRCA2	rs28897727	TGGCCAGTTTATGAAGGAGGG	GGAAAAGTTATGCAATTCTTCTGGT	277
BRIP1	rs4988345	TGGCATTAATACATACTTTCTGTGG	GTTGTAATGAGGTGCTTATTTGCAT	310
ERCC2	rs142568756	AATGAAGCTGACATAGCGGTG	CTAAGACAGAGAAGGGAGGAGGA	257
ERCC3	rs145201970	CAGCTGTGGGCTTTAGGTCA	CCGGTTGTTGACTGAGCAAG	264
MRE11	rs777373591	AGGCATGCTTTCCACAGACA	TGCAGTTTGCCTATGATTGCATTA	261
MSH6	rs142254875	ACTTAGGCTGATAAAACCCCCAAA	GCTCCTCTTCCTCACAGCCTA	251
MUTYH	rs36053993	CATCCTTGGCTATTCCGCTG	ACCTGGATACTGGGCGTG	268
PARP2	rs200603922	CCTGTCCCTCACAGCCATCTTC	AGGTTATAGGGAGCTGGAAGGG	264
PMS2	rs1554304601	TGGCAGCGAGACAAAACAGA	TCCTTACTTTACACTCTCTTTCAGC	265
POLE	chr12:133219216	GTGCTCACCTGCTCATCTCG	GGTCCACCCAGGTCTTTTCT	272
POLE	rs36120395	CAACGCAGCCCAGTAAGAAC	TTTGAACTTGCCCCCATTGC	254
POLE	rs41561818	GGCGGGTTTCTTTCCTCCAT	GACTCCGAATAGCGTGTGCT	299
PTEN	rs773513402	GGTAAGAAACACAGCAACAATGAC	GCAGCACATGAAGCATCCAC	254
PTEN	rs587779989	TGGGACGCGACTGCG	GGCTGCACGGTTAGAAAAGAC	251
RAD51C	rs61758784	TATTCTTGGGGGTGGAGTGC	TGTTTCTTTTGCAAATTGTACTGCA	310
RECQL4	rs780723602	CCTGATTCTCCAACCTCGTCT	TGAGCGGGCACTCCCAATA	305

Supplementary Table S6: TaqMan™ genotyping assay information

Gene	Variant	TaqMan™ genotyping assay (ThermoFisher Scientific)
BARD1	rs3738888	C__27471488_10
BRCA2	rs28897727	C__11711256_20
BRIP1	rs4988345	C__2649849_20
ERCC3	rs145201970	C_168471925_10
MUTYH	rs36053993	C__27860252_10
PARP2	rs200603922	C_190044127_10

Supplementary Table S7: Prostate cancer status of variant carriers

Gene	Variant	Tasmanian Familial Prostate Cancer Study (Discovery Families)					*PROGRESS*		Total
		Familial Cases	Unaffected Male Relatives	Female Relatives	Sporadic Cases	Sporadic Controls	Familial Cases	Unaffected Male Relatives	
BARD1	rs3738888	8 (2)	5 (3)	4 (0)	10	4	4	0	35
BRCA2	rs28897727	6 (3)	7 (5)	4 (1)	7	0	4	1	29
BRIP1	rs4988345	7 (2)	1 (1)	6 (2)	9	2	6	0	31
ERCC3	rs145201970	5 (2)	4 (4)	1 (1)	3	3	5	0	21
MUTYH	rs36053993	2 (2)	5 (2)	7 (1)	4	5	9	0	32
PARP2	rs200603922	6 (4)	3 (1)	1 (1)	4	0	2	0	16

Supplementary Table S8: Clinical characteristics of affected familial DDR variant carriers

Individual ID	Genotype	Population	Age at Diagnosis	Gleason Score
***BRIP1* rs4988345**				
PC2-2	GA	Australian	53	5 (3+2)
PC2-13	GA	Australian	54	n.a.
PC22-239	GA	Australian	64	7 (3+4)
PC55-309	GA	Australian	74	7 (3+4)
PC55-315	GA	Australian	74	6 (3+6)
PC3256-123	GA	Australian	61	7 (3+4)
PC3256-174	GA	Australian	73	9 (4+5)
7-700	GA	North American	63	n.a.
19-427	GA	North American	66	5 (2+3)
32-178	GA	North American	68	n.a.
70-379	GA	North American	72	8 (4+4)
120-697	GA	North American	63	8 (3+5)
133-275	GA	North American	72	7 (3+4)
***ERCC3* rs145201970**				
PC4-1	GA	Australian	60	6 (3+3)
PC4-4	GA	Australian	71	n.a.
PC12-2	GA	Australian	81	7 (4+3)
PC22-7	GA	Australian	61	n.a.
PC2494-199	GA	Australian	73	6 (3+3)
6-573	GA	North American	70	5 (2+3)
6-580	GA	North American	62	7 (3+4)
45-677	GA	North American	66	n.a.
45-718	GA	North American	54	8 (4+4)
64-623	GA	North American	65	7 (2+5)
***MUTYH* rs36053993**				
PC22-239	CT	Australian	64	7 (3+4)
PC72-4	CT	Australian	70	9 (4+5)
46-922	CT	North American	54	8 (4+4)
76-996	CT	North American	77	6 (3+3)
112-221	CT	North American	60	n.a.
112-770	CT	North American	67	5 (2+3)
112-865	CT	North American	68	7 (3+4)
112-896	CT	North American	73	n.a.
114-097	CT	North American	54	7 (3+4)
114-319	CT	North American	69	8 (3+5)
114-326	CT	North American	63	6 (3+3)
***PARP2* rs200603922**				
PC1-304	AG	Australian	70	n.a.
PC2-1	AG	Australian	51	10 (5+5)
PC2-3	AG	Australian	58	n.a.
PC2-21	AG	Australian	72	9 (4+5)
PC2-27	AG	Australian	52	5 (3+2)
62-179	AG	North American	73	8 (5+3)
66-003	AG	North American	63	n.a.

n.a. = Not Available

Supplementary Table S9: DDR variant rates by age at diagnosis

	PrCa cases meeting criteria N (%)		Variant carriers meeting criteria* N (%)	
**Total PrCa Cases**	1159		81	
**Age at Diagnosis <50 years**				
All PrCa cases	29	2.50%	2	6.90%
DDR variant carriers	2	2.47%		
**Age at Diagnosis <55 years**				
All PrCa cases	117	10.09%	11	9.40%
DDR variant carriers	11	13.58%		
**Age at Diagnosis <60 years**				
All PrCa cases	344	29.68%	20	5.81%
DDR variant carriers	20	24.69%		
**Age at Diagnosis <65 years**				
All PrCa cases	693	59.79%	38	5.48%
DDR variant carriers	38	46.91%		
**Age at Diagnosis <70 years**				
All PrCa cases	975	68.59%	62	6.36%
DDR variant carriers	62	76.54%		

*Values represent the number and percentage of Tasmanian and North American cases diagnosed within the specified age bracket who carry a rare DDR risk variant.
